# Computational Study on the Mechanism of the Photouncaging Reaction of Vemurafenib: Toward an Enhanced Photoprotection Approach for Photosensitive Drugs

**DOI:** 10.3390/molecules26071846

**Published:** 2021-03-25

**Authors:** Abdulilah Dawoud Bani-Yaseen

**Affiliations:** Department of Chemistry & Earth Sciences, Faculty of Arts & Science, Qatar University, Doha 2713, Qatar; abdulilah.baniyaseen@qu.edu.qa

**Keywords:** vemurafenib, photoprotected prodrug, photouncaging mechanism, DFT/TD-DFT, NBO

## Abstract

The photochemical behavior of the photosensitive first-line anticancer drug vemurafenib (VFB) is of great interest due to the impact of such behavior on its pharmacological activity. In this work, we computationally elucidated the mechanism of the photoinduced release of VFB from the 4,5-dimethoxy-2-nitrobenzene (DMNB) photoprotecting group by employing various density functional theory (DFT)/time-dependent DFT (TD-DFT) approaches. The computational investigations included a comparative assessment of the influence of the position of the photoprotecting group as a substituent on the thermodynamics and kinetics of the photouncaging reactions of two VFB-DMNB prodrugs, namely pyrrole (N_P_) and sulfonamide (N_S_). With the aid of the DFT calculations concerning the activation energy barrier (∆G^‡^), the obtained results suggest that the step of the photoinduced intramolecular proton transfer of the DMNB moiety is not detrimental concerning the overall reaction profile of the photouncaging reaction of both prodrugs. However, the obtained results suggested that the position of the substitution position of the DMNB photoprotecting group within the prodrug structure has a substantial impact on the photouncaging reaction. In particular, the DMNB-N_s_-VFB prodrug exhibited a notable increase in ∆G^‡^ for the key step of ring opining within the DMNB moiety indicative of potentially hindered kinetics of the photouncaging process compared with DMNB-N_p_-VFB. Such an increase in ∆G^‡^ may be attributed to the electronic influence of the N_P_ fragment of the prodrug. The results reported herein elaborate on the mechanism of the photoinduced release of an important anticancer drug from photoprotecting groups with the aim of enhancing our understanding of the photochemical behavior of such photosensitive pharmaceutical materials at the molecular level.

## 1. Introduction

Examining the physicochemical properties of pharmaceutical materials at the molecular level can enhance our understanding of various associated biological processes that are important in drug discovery. Examining such molecular properties of pharmaceutical materials has recently attracted notable attention [1,2,3,4,5,6,7,8,9,10,11,12,13]. The phototoxicity of numerous pharmaceutical materials is a major therapeutic concern that can conspicuously constrain their clinical applications [14,15,16,17,18]. Several approaches have been recently developed toward reducing the impact of such undesired side effects, including photocaging [19,20,21,22,23,24]. Recently, the importance of the incorporation of photocaging groups into photoactive pharmaceutical materials has been increasingly recognized as an effectual approach toward reducing the phototoxicity of a wide spectrum of photoactive drugs [20,21,22,23,24]. 

In such an approach, a photoremovable photocaging moiety is selectively attached to specific positions of a molecule of interest via covalent bonding to afford a photocaged product of rendered photochemical activities. Subsequently, the functionality of the parent molecules can be released upon the elimination of the photocaging group via a photochemical process [25,26,27]. Such a process of uncaging is substantially important in the landscape of pharmaceutical and medicinal chemistry, where it requires irradiation with light of a specific wavelength (λ_irr_) that is appropriate for uncaging the drug under study. 

As such, several studies have been recently reported concerning the photocaging of materials of biological and pharmaceutical importance, such as the antimelanoma agent vemurafenib [20], anticancer drug doxazolidine [21], anticancer kinase inhibitor imatinib [22], small-molecule tubulin inhibitor [23], and photocaged materials for homeostasis [24]. Various types of photolabile-protecting groups (PPGs) have been developed and utilized in these works to afford the photocaged prodrugs of interest. Such photocaging groups have been mainly utilized to selectively photoprotect specific functional groups that are feasible for photoreactions; these include amines, hydroxyl, and carboxyl functional groups. 

**Figure 1:**
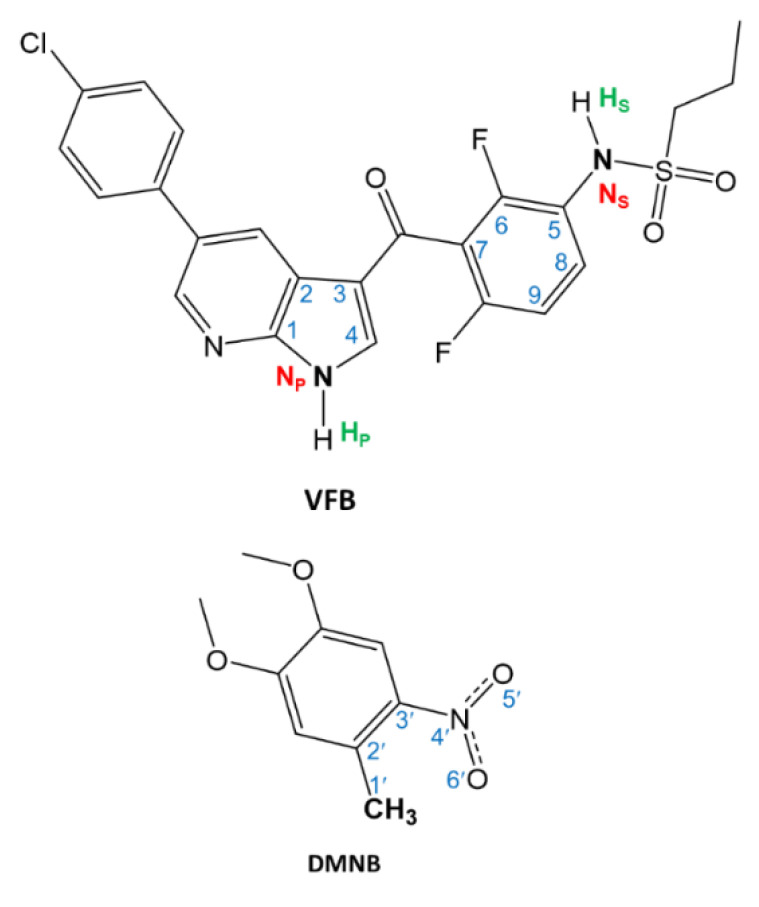
The chemical structures of vemurafenib (VFB) and 4,5-dimethoxy-2-nitrobenzene (DMNB).

Interest in the applications of the antimelanoma agent vemurafenib (VFB) has recently grown. The chemical structure of VFB is shown in Figure 1. VFB has attracted such notable attention due to its therapeutic efficacy as an anticancer drug. However, although VFB is considered as a first-line anticancer drug, several studies have reported the severe phototoxicity associated with the administration of the drug [28,29,30,31,32,33]. On the other hand, investigating the physicochemical properties of materials of biological and medicinal applications at the molecular level is essential for a better understanding of their activities and functionalities [21,22,23,24,25,26,27,28,29,30,31]. As such, investigating the photochemical properties of photosensitive biological materials, such as the first-line anticancer drug VFB, at the molecular level is necessary to enhance the efficacy of the photocaging process and, consequently, reduce the potential phototoxicity. 

**Scheme 1:**
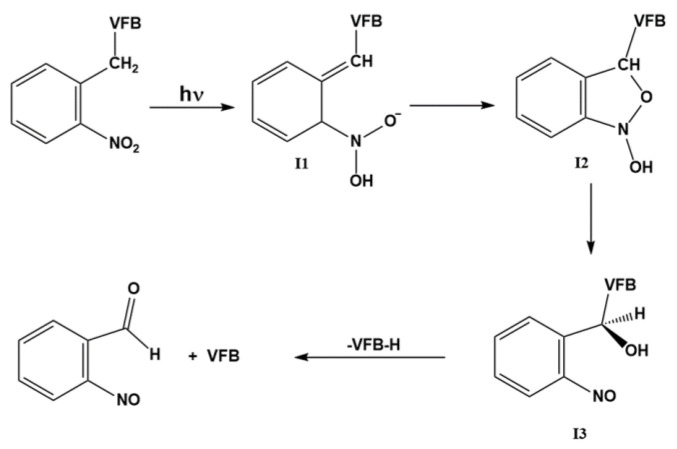
Mechanism of the photouncaging reaction of VBF protected by DMNB.

In examining the structure, VFB bears two secondary amine functional groups that are feasible for photochemical instability. These two amines are within two moieties, namely the azaindole and sulfonamide moieties. Employing the photocaging approach for these two photoactive centers toward reducing the phototoxicity of VFB was recently reported by Peifer and co-workers using derivatives of 2-nitrotoluene, such as 4,5-dimethoxy-2-nitrobenzyl (DMNB) [20]. The reported results suggested a promising approach for photocaging and photouncaging of VFB using the DMNB moiety. However, a requested feature that is expected from an efficient photocaging process of biological materials is a reversible uncaging of the photoactive drug under ambient conditions and appropriate microenvironments. Importantly, such requirements necessitate investigating various mechanistic aspects of the uncaging process under biologically mimicked microenvironments. In the literature, there are several studies on the mechanism of photouncaging of 2-nitrotoluene derivatives [20,25,34,35,36]. In view of the suggested mechanisms in these references, a plausible mechanism for the photocleavage reaction of VFB from DMNB can be suggested, as illustrated in Scheme 1.

In light of the above-mentioned considerations, we provide, in this work, computational insights concerning selected mechanistic aspects of the photouncaging reaction of the DMNB-photoprotected version of the first-line anticancer drug VFB. By employing systematic density functional theory (DFT) calculations in implicit aqueous solutions, we address important scientific concerns at the molecular level related to the mechanism of the photouncaging reaction of VFB photoprotected by DMNB, which is induced by irradiation. The results presented in this work can help enhance our understanding of this important chemical process toward the development of efficient chemical approaches that can lead to reducing the phototoxicity of photosensitive drugs.

## 2. Results and Discussion 

**Figure 2:**
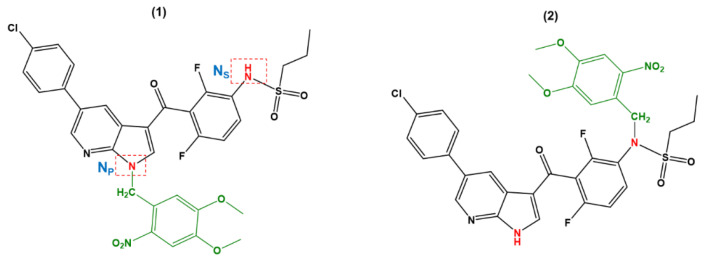
Chemical structures of the DMNB-photocaged VFB pyrrole (N_P_) and sulfonamide (N_S_) amine groups considered in this work.

As can be noted from Figure 1, the structure of VFB bears two amine groups that are feasible for photoprotection with DMNB. These two amine groups are ascribed as pyrrole (N_P_) and sulfonamide (N_S_). These are ascribed with the correspondence of the moieties that bear these two amine groups, namely the sulfoxide and pyrrole moieties, respectively. Figure 2 displays the chemical structures of the DMNB-photocaged VFB prodrugs, namely **1** and **2**. The ascriptions of **1** and **2** correspond to the photoprotected VFB at the N_P_ and N_S_ positions, respectively.

According to the mechanism illustrated in Scheme 1, the photouncaging reaction of the DMNB-protected substance is triggered by irradiation that induces a proton transfer in the excited state to afford **I1**. Such a step is plausibly considered as a fast step that has a minimal influence on the overall kinetics of the photouncaging reaction [34,35,36]. As such, this particular step was not considered in the DFT calculations concerning the transformation of **1** and **2** into their corresponding **I1**. Hence, we focused on the other three steps in this study. The other three steps of the photouncaging reaction can be briefly described. As such, following the photoinduced intramolecular proton transfer in **1** and **2**, **I1** undergoes an intramolecular cyclization reaction between C′′_1_ and O′_6_ to afford **I2**. Then, **I2** undergoes a concurrent a ring-opening and proton transfer to yield **I3** followed by the breakage of the N-C′_1_ bond to release the parent drug VFB. We also examined the transition state (TS) for each of these three steps of the photouncaging of **1** and **2** as well.

**Figure 3:**
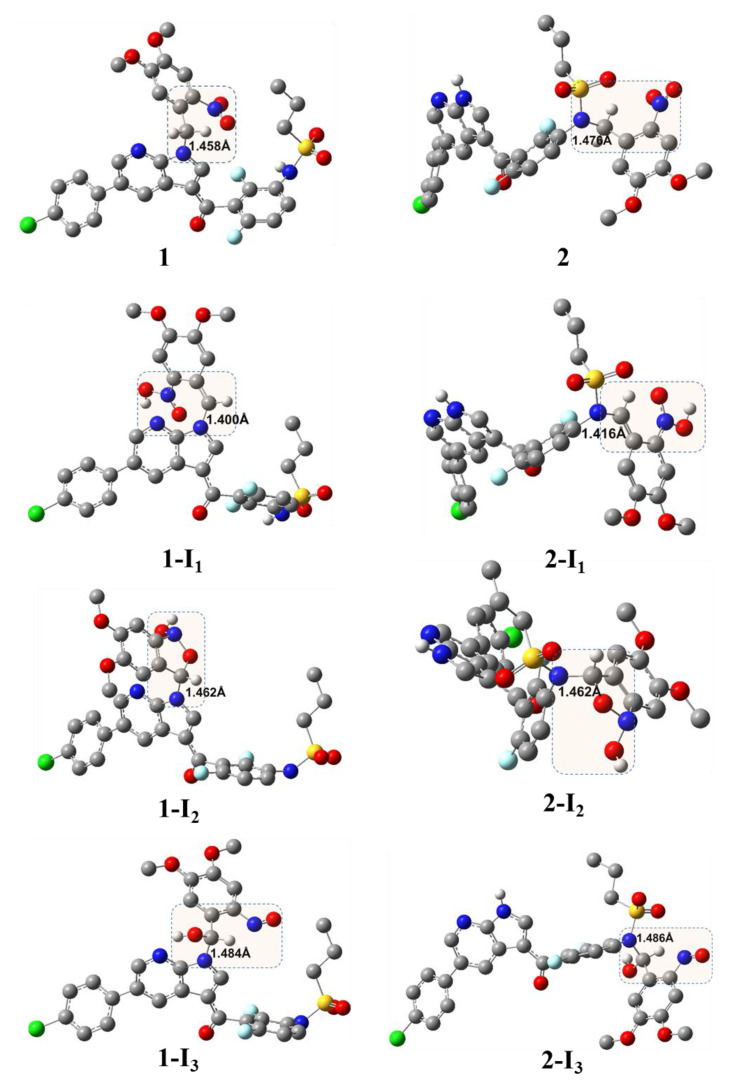
DFT/B3LYP/6-31+G(d)-optimized geometry of the DMNB-photocaged VFB and their corresponding photouncaging intermediates; uninvolved hydrogen atoms were omitted for clarity; highlighted regions indicate the center of the reaction; numbers indicate the bond length in Å.

Geometry optimization. Figure 3 shows the DFT-optimized geometry of **1** and **2** and the corresponding intermediates that are generated via the photouncaging reaction. The accuracy of the optimized geometry was verified upon comparison with the previously reported X-ray structures obtained from the Cambridge Crystallographic Data Centre (CCDC). The key structural parameters of the examined species, namely the bond lengths of N_P_-C′_1_ and N_S_-C′_1_ are displayed in Figure 3. Comparing **1** vs. **2**, the calculations revealed a shorter bond length for **1** with a ∆l of 0.018 Å. Additionally, comparing the corresponding intermediates of **1** vs. their counterparts of **2**, this variation in the l_(N-C′)key_ was retained for **I1**, whereas no significant differences were noted for **I2** and **I3**. On the other hand, upon examining the optimized geometry of **1** and **1-I1**, there was substantial change in the dihedral angle that comprises the N_P_-C′_1_ and the DMNB moiety. Hence, the formation of **1-I1** requires a ~100° rotation in this dihedral angle of the DMNB moiety around the N_P_-C′_1_ bond axis. This kind of rotation requirement may increase the energy barrier for the **1**→**1-I1** transformation, which in turn might be crucial for affording the photouncaging process of 1. The relative rotation requirement around the key-bond of the photouncaging reaction for **I2** and **I3** of both prodrugs is not significantly appreciable.

**Figure 4:**
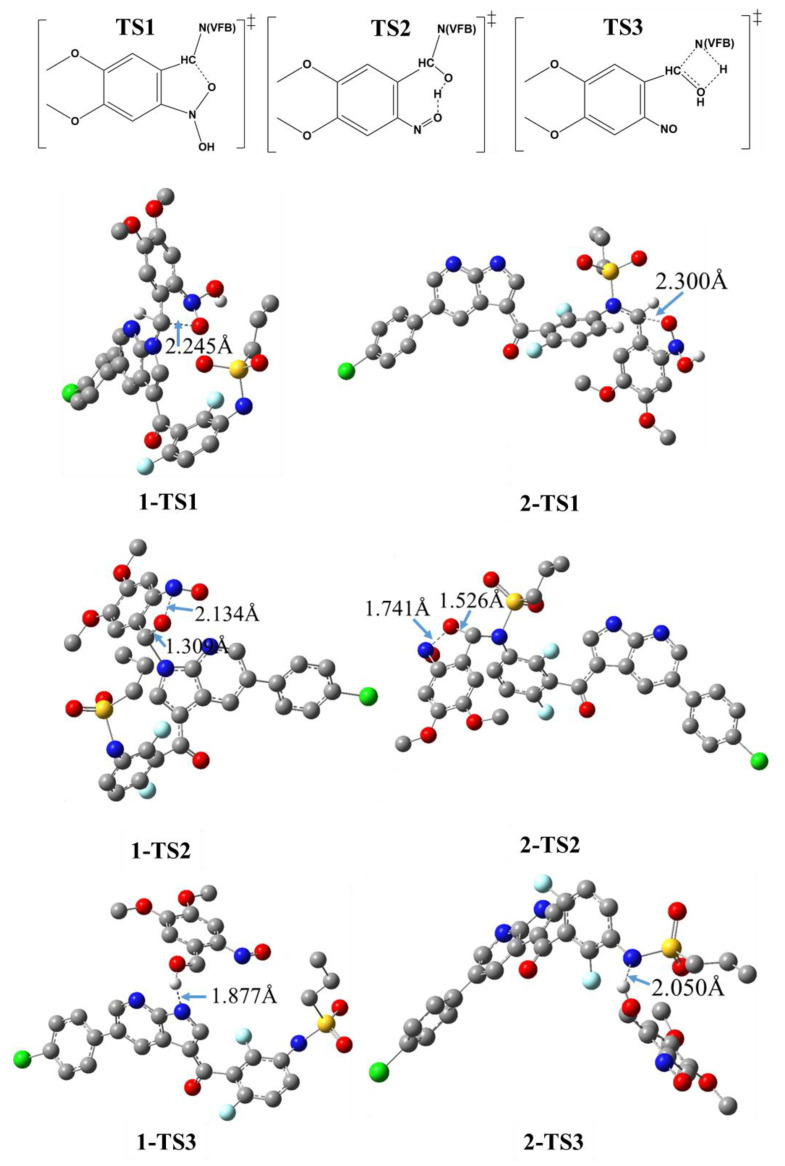
DFT/B3LYP/6-31+G(d))-optimized geometry of the transition state (TS) of the photouncaging of **1** and **2**; uninvolved hydrogen atoms are omitted for clarity; the given numbers correspond to the bond length of selected key bonds as indicated by arrows.

According to the mechanism illustrated in Scheme 1, we performed a geometry optimization of TSs for the three critical steps following the intramolecular proton transfer induced by irradiation employing the DFT method with the 6-31G basis set (DFT/6-31G) in a vacuum. The optimized geometry of **TS1-3** of the photouncaging of **1** and **2** are displayed in Figure 4. 

Figure 4 illustrates the distance (d_A…B_) in Å between the two key atoms involved in the key TS of the photouncaging reactions of **1** and **2**. As stated above, **TS1** refers to the transformation **I1**→**TS1**→**I2**, which corresponds to the formation of the C′_1_-O′_6_ bond ascribed as an intramolecular cyclization reaction. Comparing the **1-TS1** vs. **2-TS2**, the DFT calculation revealed a d_C′1…O′6_ of 2.245 and 2.300 Å, respectively, revealing a difference of 0.055 Å. Such a small increase in the distance observed for **1-TS1** compared to **2-TS1** is indicative of the relatively comparable energy barrier that needs to be overcome for both intermediates. For the **I2**→**TS2**→**I3** transformation, we found a d_N′4…O′6_ of 2.134 and 1.741 Å for **1-TS2** and **2-TS2**, respectively, indicative of a relatively more feasible ring-opining for **1-TS2** compared to **2-TS2**. As such, one may anticipate that a higher energy barrier is associated with the formation of **2-TS2** compared to **1-TS2**. 

Studying the photochemical behavior of a substance necessitates investigating the potential electronic transition associated with being irradiated with light of specific intensity and λ. On the other hand, for safe and efficient pharmacological applications, reducing the damage to the tissues that is caused by the irradiation of the uncaging process can be achieved by increasing the effective λ of the irradiation process. One important aspect of the photouncaging mechanism of the **1** and **2** prodrugs that we consider herein is to assess their photochemical behavior upon being irradiated with the same light source of specific λ. 

**Figure 5:**
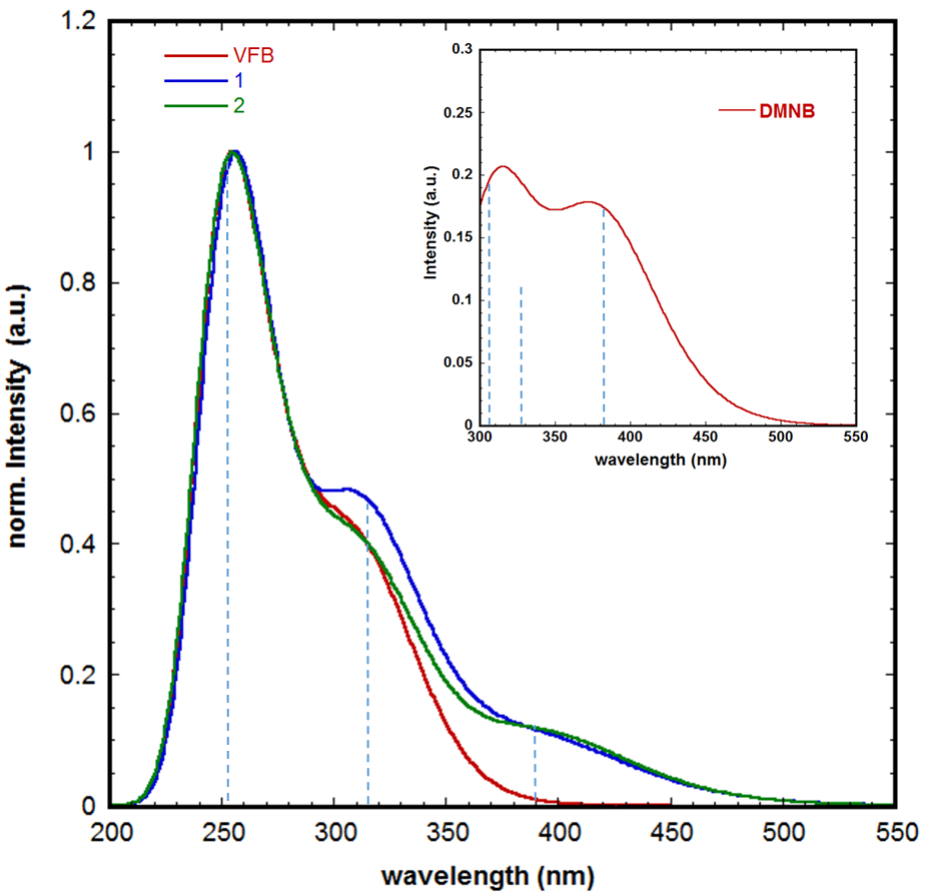
Time-dependent DFT (TD-DFT) (B3LYP/6-31 + G(d))-simulated UV–Vis absorption spectra of VFB in implicit aqueous solution; inset: simulated spectrum of DMNB under the same conditions; vertical lines indicate the position of the key electronic transition that is accountable for the corresponding absorption band.

Thus, investigating their spectral behavior is crucial toward providing better comparative rationalization of such potential discrepancies in their photochemical behaviors. As such, we used the time-dependent DFT (TD-DFT) approach to compute the UV–Vis absorption spectra of the parent compounds, namely VFB and DMNB, as well as prodrugs **1** and **2** in implicit aqueous solutions (Figure 5). First, the accuracy of the calculated spectra of VBF, **1**, and **2** was verified by comparing the simulated spectra vs. the experimental counterparts [20,37]. As can be noted from Figure 5, VFB exhibits two major absorption bands positioned at λ of 253 and 315 nm. For **1** and **2**, a third major absorption band appeared in the visible range at a λ of ~ 390 nm. However, comparing the absorption spectrum of VFB vs. DMNB, **1** and **2**, one can infer that the absorption band of λ_390_ corresponds to the DMNB moiety. 

**Figure 6:**
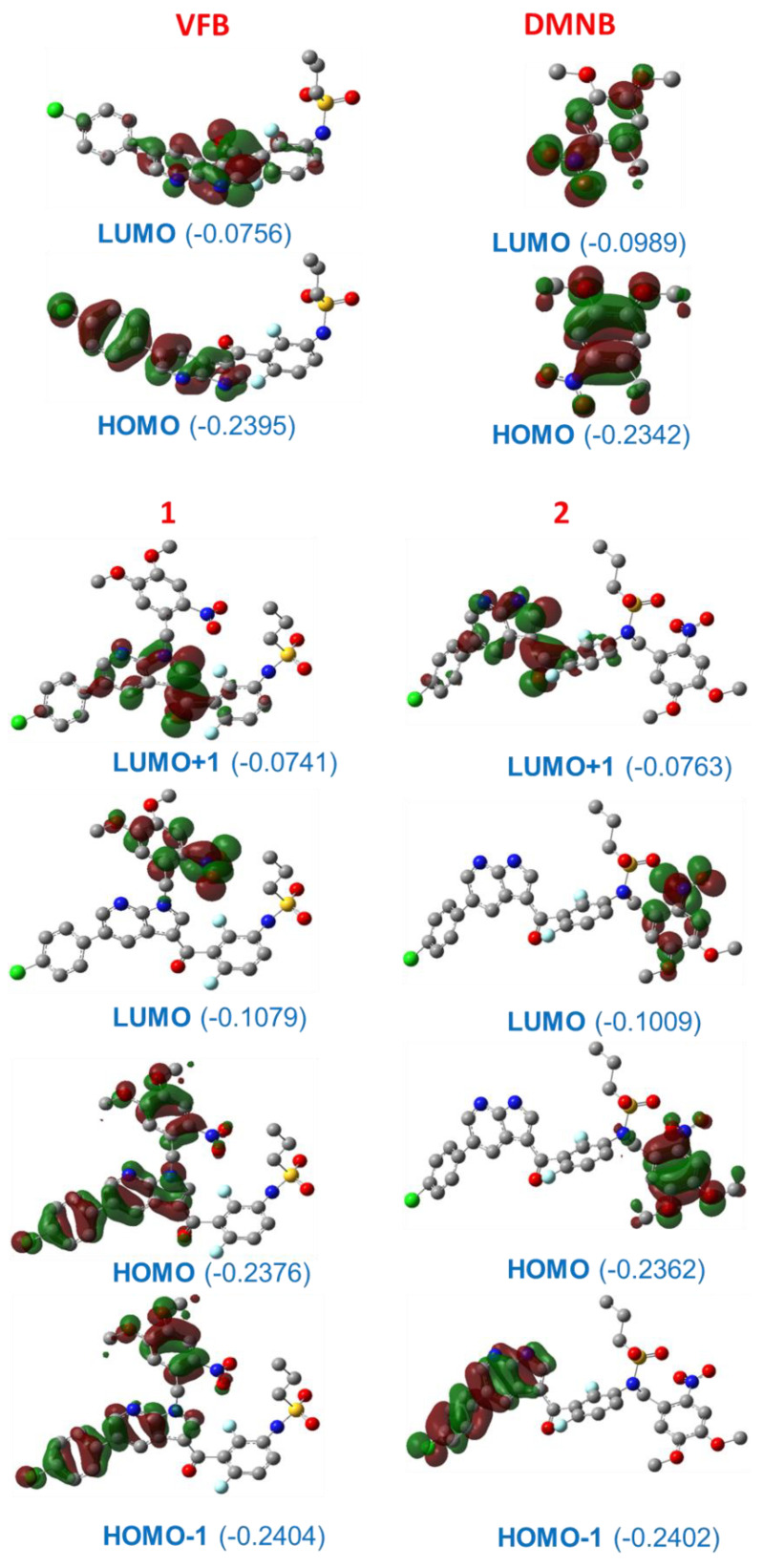
The frontier molecular orbitals (Mos) of VFB, DMNB, **1**, and **2**; numbers in brackets refer to energy in hartree; uninvolved hydrogen atoms are omitted for clarity.

The TD-DFT results revealed that all three major transition bands illustrated in Figure 5 correspond to π→π* electronic transitions. The key molecular orbitals (MOs) involved in such transitions are displayed in Figure 6. For VFB, both the highest molecular occupied orbital (HOMO) and lowest unoccupied molecular orbital (LUMO) are delocalized over the π-conjugated part of the molecule comprising the phenyl, pyridine, and pyrrole moieties. The calculations revealed an energy gap (∆E_HOMO-LUMO_) of 4.46 and 3.68 eV for VFB and DMNB, respectively. One can anticipate that the lone-pair (lp) of the N_P_ can participate in the charge delocalization that accounts for the shape and nature of these frontier MOs.

Comparing the MOs of VFB vs. **1** and **2**, we infer that the HOMO and LUMO of VFB become a HOMO-1 and LUMO+1 in **1** and **2**, respectively, whereas the HOMO and LUMO of **1** and **2** correspond to their counterparts of DMNB. However, the incorporation of DMNB into VFB via a photocaging reaction induced an increase in the ∆E of the MOs of 0.065 eV. This increase in the ∆E corresponds to the stabilization and destabilization of 0.025 and 0.040 in the energy of HOMO and LUMO, respectively, indicative of a blue-shift in the electronic transition and consequently reduced photoreactivity in the visible range of irradiation. Comparing the HOMO of **1** vs. the counterpart of **2**, the HOMO comprises both HOMOs of VFB and DMNB, whereas for **2**, it comprises only the HOMO of DMNB. In light of these changes in the nature of the MOs after photocaging, we suggest that **1** has to overcome higher energy barriers concerning the photouncaging reaction, which in turn is well in line with the results obtained from the DFT calculations obtained from the optimized geometry.

We further extended our investigations concerning the role of the position of photoprotection on the photouncaging reaction by conducting a natural bond orbital (NBO) analysis. In this approach, the contributions of N_P_ and N_S_ in the mechanistic pathway of **1** and **2** were evaluated. We focused herein on the energy of interaction between the MOs of the key atoms involved in the photouncaging reaction. Such types of interactions are presented in terms of an electronic transition from a donating MO to an accepting MO; i.e., D_MO_→A_MO_. The magnitude of interaction was calculated with respect to the second-order perturbation energy (E(2)) as revealed by the DFT-based NBO analysis. 

**Figure 7:**
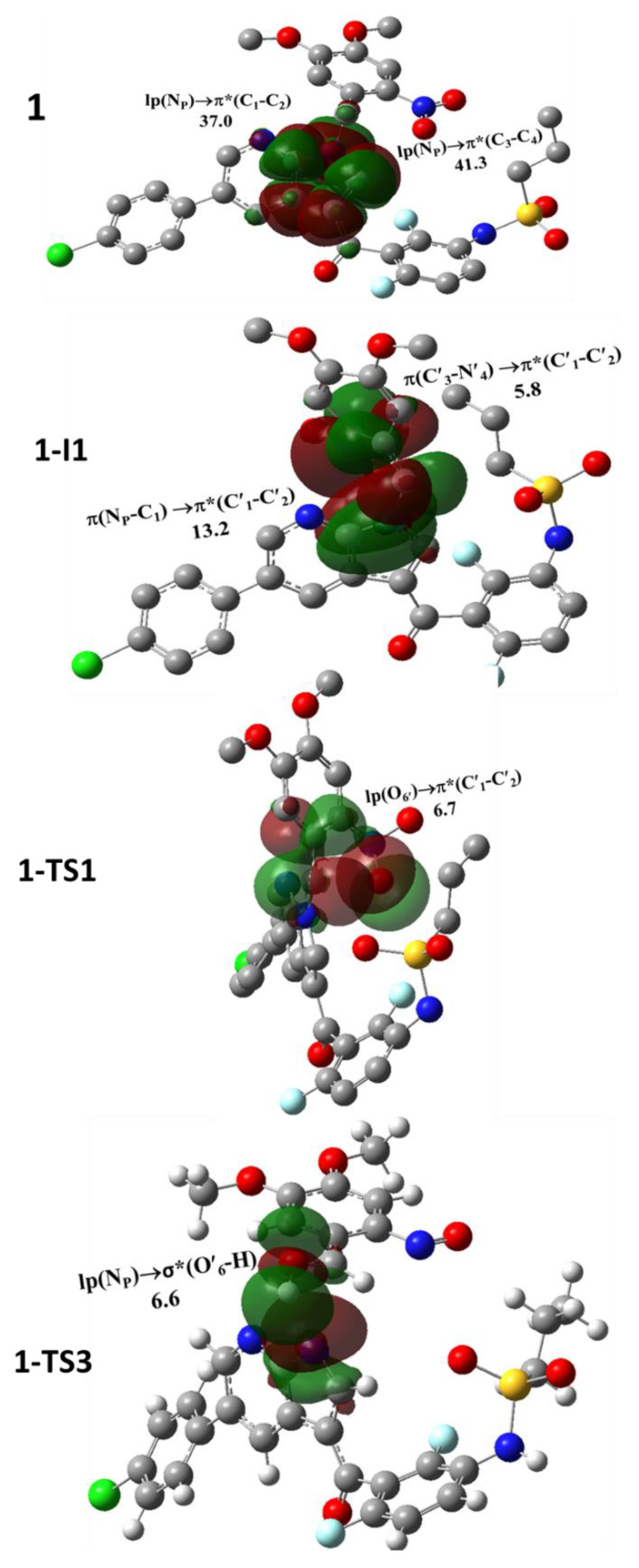
Superposition of NBOs of selected species; uninvolved hydrogen atoms are omitted for clarity.

The obtained results from the NBO analyses of all species involved in the photouncaging reaction of **1** and **2** are compiled in Table 1. The superpositions of the selected NBO and the corresponding energy of interactions are displayed in Figure 7. It is worth noting that N_P_ and N_S_ in VFB exhibited notable NBO discrepancies. N_P_ exhibited a substantial two electron transition of the types lp(N_P_)→π*(C_1_-C_2_) and lp(N_P_)→π*(C_3_-C_4_) with E(2) of 42.0 and 35.9 kcal/mol, respectively; whereas N_S_ exhibited two transitions of the types lp(N_S_)→σ*(S-O) and lp(N_S_)→σ*(C_5_-C_6_) with E(2) of 8.2 and 7.8 kcal/mol, respectively. These results revealed strong interactions between the lp(N_P_) and adjacent MOs indicative of enhanced charge delocalization, which in turn indicates a reduced contribution in the photouncaging reaction. 

As such, we suggest that the lp(N_S_) can afford more contribution toward promoting the photouncaging reaction for **2** compared with **1**. The formation of **1-I1** can trigger a substantial deformation in the delocalization of the N_P_ moiety. In particular, upon the formation of **1-I1**, a loss in the lone-pair character of N_P_ is observed. Under these circumstances, the N_P_ atom exhibits other types of stabilizations, namely π(N_P_-C_1_)→π*(C′_1_-C′_2_) with E(2) of 13.2 kcal/mol indicative of charge delocalization that is well in line with the frontier HOMO of prodrug **1**. These results suggest that the change in the types of NBO electronic transition may cause a diminution in the relative reactivity of **1** compared with **2** toward the formation of **I1**. 

The lp(N_P_) retained its characteristic upon the formation of **I2** and **I3**. On the other hand, the main transitions of lp(N_S_) retained their characters, including the two main transitions. Concerning the transition between the lp(N_P_) and lp(N_S_) to the antibonding NBO of the key atoms of the DMNB moiety, namely σ* and π*, we suggest that such types of transition can promote bond formation breakage toward affording the species of interest. For example, **1-I2** and **2-I2** exhibited the transitions of lp(N_P_)→σ*(C′_1_-O′_6_) and lp(N_S_)→σ*(C′_1_-O′_6_), respectively, with stabilizing energies of 16.1 and 17.6 kcal/mol, respectively. These results suggest that such transitions can stimulate the breakage of the C′_1_-O′_6_ bond and consequently the ring-opening and proton transfer toward affording **I3**. Indeed, alike transitions can be noted in the **TS2** of both intermediates indicative of the important contribution of lp(N_P_) and lp(N_S_) in the photouncaging reaction of **1** and **2**.

We further extended our NBO analyses via examining the variation in the NBO charges of the key atoms involved in the photouncaging reaction of prodrugs **1** and **2**; see Table 2. As anticipated, N_P_ and N_S_ exhibited negative charges for all species that can be attributed to the higher electronegativity of the nitrogen atom. However, comparing the N_P_ vs. the N_S_ for VFB, the calculations revealed NBO charges of −0.534 and −0.869 e, respectively. This relatively lower value for N_P_ compared to N_S_ can be attributed to the incorporation of the lp(N_P_) in the conjugation system as demonstrated by the frontier MO and NBO analyses.

However, both exhibited relatively similar behaviors concerning the decrease in their values upon the formation of the prodrugs **1** and **2**, where a decrease in the NBO charge by 0.175 and 0.202 e was calculated for **1** and **2**, respectively. On the other hand, N_P_ and N_S_ exhibited relatively negligible changes in their NBO charges in the corresponding intermediates and TSs. Importantly, the key atom C′_1_ exhibited a substantial variation in its NBO charge as the photouncaging reaction progressed. For example, C′_1_ exhibited approximately the same values of NBO charges for **1** and **2**, whereas values of 0.019 and −0.005 were calculated for **1-I1** and **2-I1**, respectively.

Similarly, for **1-TS1** and **2-TS1**, values of 0.123 and 0.057 were calculated for the NBO charges, respectively, indicative of an ∆NBO charge of 0.066 e. Nevertheless, other species of **1** compared to **2** exhibited an insignificant type of ∆NBO. Indeed, these results indicate the significant influence of the first step, namely irradiation-induced prodrug→**I1**, in distinguishing the photochemical behavior of the prodrugs **1** and **2**. Such observations were further illustrated as shown in the overall energy profile of the photouncaging reactions of **1** and **2**.

**Figure 8:**
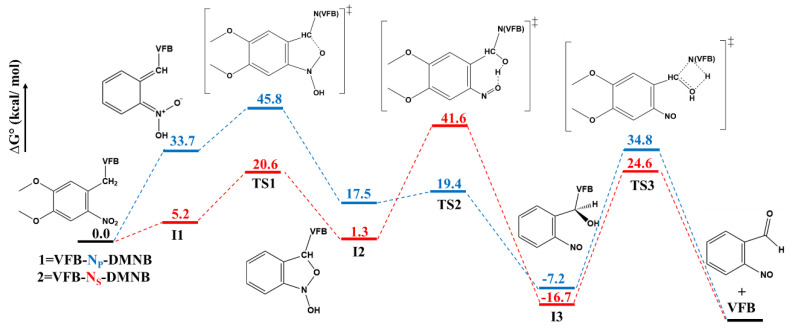
Energy profile of the photouncaging reactions of **1** and **2**. The Gibbs energy of the examined species was obtained from geometry optimization at the DFT/B3LYP /6-31G level of theory.

Reaction Profile. As illustrated in Figure 8, we constructed the overall energy profile for the photouncaging reaction of **1** and **2** with the aid of DFT/B3LYP /6-31G calculations and took into consideration the corresponding key steps. The overall energy was constructed in terms of the relative change Gibbs free energy (∆G°) utilizing the results of the geometry optimization of intermediates and TSs. The displayed results were calculated relative to the energy of the reactants, namely prodrugs **1** and **2**. For example, the value of 33.7 kcal/mol indicates the difference in Gibbs free energy between **1-I1** and **1**; i.e., G°(**1-I1**) − G°(**1**).

As can be inferred from Figure 8, the DFT calculations revealed a ∆G° of 33.7 and 5.2 kcal/mol for the formation of **1-I1** and **2-I1**, which is indicative of thermodynamically more favorable formation for **2-I1** compared with **1-I1** upon irradiation. This notable difference is indicative of a relatively more favorable photo-conversion of **2** compared to **1**, which is in good agreement with the experimental results reported for these two prodrugs [20]. One can infer from Figure 8 that the **I1**→**TS1**→**I2** transformations of both prodrugs are associated with comparable energy barriers of activation (∆G^‡^), where the ∆G^‡^ values of 12.1 and 15.4 kcal/mol were calculated for the transformations of **1** and **2**, respectively, which is in line with the results obtained from the geometry optimization.

On the other hand, one significant difference can be noted for the **I2**→**TS2**→**I3**. ∆G^‡^ values of 1.9 and 40.3 kcal/mol were calculated for the transformations of **1** and **2**, respectively. We suggest that such a significant increase in the ∆G^‡^ for **2** compared to 1 might be attributed to both steric and electronic effects, as revealed by the geometry optimization calculations and NBO analyses. For the termination transformation, **I3**→**TS3**→**Pr**, ∆G^‡^ values of 42.0 and 41.3 kcal/mol can be inferred from Figure 8 for **1** and **2**, respectively, indicative of similar kinetic favoritism.

These results suggest that the intramolecular proton transfer step induced by irradiation is the key thermodynamic process that is responsible for discrepancies in the photochemical behavior observed for prodrugs **1** and **2**. On the other hand, the second step is a key source of kinetic discrepancies between the two prodrugs, whereas the third step for both prodrugs exhibited a comparable increase in ∆G^‡^ compared to the other steps. These results also suggest that both thermodynamic and kinetic factors can notably affect the photouncaging reactions of prodrugs **1** and **2**.

## 3. Computational Methods

All calculations were performed with Gaussian 09 version D.01 [38]. Calculation of the Geometry optimization was conducted for all species comprised in the mechanism shown in Scheme 1 employing the DFT/B3LYP/6-31+G(d), unless otherwise noted. The optimized geometry was verified as minima utilizing frequency calculations. The optimized geometries for selected species were utilized as inputs to perform further computational tasks. The UV–Vis absorption spectra (company, city, abbreviated state of the parent compounds and prodrugs were simulated utilizing time-dependent DFT (TD-DFT) in implicit aqueous solutions. The implicit aqueous solution was incorporated into the DFT calculations employing the integral equation formalism polarizable continuum model (IEFPCM) [39]. Natural bond orbital (NBO) analyses were performed as implemented in Gaussian 09. The DFT method employed herein has been previously demonstrated to be efficient in reproducing the experimental results of the molecular properties of various substances in implicit solutions [8,40].

## 4. Conclusions

The photocaging of photosensitive pharmaceutical materials is a promising approach in pharmaceutical and biological applications toward the reduction in the potential associated phototoxicity of a drug molecule of interest. However, it is crucial to elucidate the mechanism of the photoinduced release of the parent molecule. The present study provides computational insights on the mechanism of the photouncaging reaction of the important first-line anticancer drug VFB from the photoprotecting group DMNB. Within a four-step mechanistic pathway, the obtained results concerning the thermodynamic factor and activation energy barrier revealed that the first step of the mechanism was not detrimental—namely, the photoinduced intramolecular proton transfer within the DMNB moiety.

We demonstrated that the position of the moiety DMNB as a substituent can have a substantial impact on the kinetics of the photouncaging reaction of VFB-DMNB prodrugs. Hence, these results suggest that the step of the concurrent ring-opening and intramolecular proton transfer might be the rate-determining step for prodrug **2** with an equivalent role for the termination step, whereas the termination step is the rate-determining step for prodrug **1**. We anticipate that the results reported herein may provide insights on the development of photocaging/photouncaging approaches for reducing the phototoxicity of photosensitive pharmaceutical materials.

## Figures and Tables

**Table 1:** Selected natural bond orbital (NBO) electronic transition of key elements ^(a)^ involved in the photouncaging reaction and their corresponding stabilization energies (E(2)).

Species	Transition (Donor→Acceptor)	E(2) kcal/mol	Species	Transition (Donor→Acceptor)	E(2) kcal/mol
VFB	lp(N_P_)→π*(C_1_-C_2_)	42.0	**2**	lp(N_S_)→σ*(S-O)	10.2
	lp(N_P_)→π*(C_3_-C_4_)	35.9		lp(N_S_)→σ*(C_5_-C_6_)	7.4
	lp(N_S_)→σ*(S-O)	8.2		lp(N_S_)→σ*(C_5_-C_8_)	7.8
	lp(N_S_)→σ*(C_5_-C_6_)	7.8		lp(N_S_)→σ*(C′_1_-C′_2_)	8.0
**1**	lp(N_P_)→π*(C_1_-C_2_)	41.3	**2-I1**	lp(N_S_)→σ*(S-O)	8.2
	lp(N_P_)→π*(C_3_-C_4_)	37.0		lp(N_S_)→σ*(C_5_-C_6_)	6.3
	lp(N_P_)→π*(C′_1_-C′_2_)	6.7		lp(N_S_)→σ*(C_5_-C_8_)	7.1
**1-I1**	π(N_P_-C_1_)→π*(C′_1_-C′_2_)	13.2		lp(N_S_)→σ*(C′_1_-C′_2_)	33.8
	π(C′_3_-N′_4_)→π*(C′_1_-C′_2_)	5.8	**2-I2**	lp(N_S_)→σ*(C′_1_-O′_6_)	17.6
**1-I2**	lp(N_P_)→σ*(C′_1_-O′_6_)	16.1		lp(O′_6_)→σ*(N_S_-C′_1_)	8.4
**1-I3**	lp(N_P_)→σ*(C′_1_-O′_6_)	5.6	**2-I3**	lp(O′_6_)→σ*(N_S_-C′_1_)	13.9
	lp(O′_6_)→σ*(N_P_-C′_1_)	8.5	**2-TS1**	lp(N_S_)→σ*(C′_1_-C′_2_)	26.1
	lp(O′_6_)→s*(H)	24.7		lp(O′_6_)→π*(C′_1_-C′_2_)	9.4
**1-TS1**	lp(N_P_)→π*(C′_1_-C′_2_)	46.9	**2-TS2**	lp(N_S_)→σ*(C′_1_-O′_6_)	22.2
	lp(O′_6_)→π*(C′_1_-C′_2_)	6.7	**2-TS3**	lp(N_P_)→σ*(O′_6_-H)	7.1
**1-TS2**	lp(N_P_)→σ*(C′_1_-O′_6_)	16.4			
**1-TS3**	lp(N_P_)→σ*(O′_6_-H)	6.6			

^(a)^ See Figure 1 for atom numbering.

**Table 2:** The NBO charges ^(a)^ of key atoms ^(b)^ of the intermediates and TSs of photouncaging reactions of prodrugs **1** and **2**.

Species	1			2		
	Np	Ns	C′_1_	Np	Ns	C′_1_
VFB	−0.534	−0.869				
prodrug	−0.359	−0.849	−0.282	−0.534	−0.667	−0.281
**I1**	−0.349	−0.836	0.019	−0.533	−0.598	−0.005
**TS1**	−0.336	−0.832	0.123	−0.533	−0.600	0.057
**I2**	−0.382	−0.836	0.231	−0.534	−0.663	0.217
**TS2**	−0.338	−0.842	0.247	−0.533	−0.658	0.232
**I3**	−0.384	−0.867	0.256	−0.533	−0.687	0.242
**TS3**	−0.591	−0.839	0.381	−0.546	−0.832	0.361

^(a)^ In units of electron (e); e = 1.6022 × 10^−19^ C. ^(b)^ See Figure 1 for atom numbering.

## Data Availability

Not applicable.

## Sample Availability

Samples of the compounds are not available from the author.
